# The Role of Flavonoids in Nodulation Host-Range Specificity: An Update

**DOI:** 10.3390/plants5030033

**Published:** 2016-08-11

**Authors:** Cheng-Wu Liu, Jeremy D. Murray

**Affiliations:** Department of Cell & Developmental Biology, John Innes Centre, Norwich, Norfolk NR4 7UH, UK; Chengwu.Liu@jic.ac.uk

**Keywords:** methoxychalcone, daidzein, genistein, medicarpin, phytoalexins

## Abstract

Flavonoids are crucial signaling molecules in the symbiosis between legumes and their nitrogen-fixing symbionts, the rhizobia. The primary function of flavonoids in the interaction is to induce transcription of the genes for biosynthesis of the rhizobial signaling molecules called Nod factors, which are perceived by the plant to allow symbiotic infection of the root. Many legumes produce specific flavonoids that only induce Nod factor production in homologous rhizobia, and therefore act as important determinants of host range. Despite a wealth of evidence on legume flavonoids, relatively few have proven roles in rhizobial infection. Recent studies suggest that production of key “infection” flavonoids is highly localized at infection sites. Furthermore, some of the flavonoids being produced at infection sites are phytoalexins and may have a role in the selection of compatible symbionts during infection. The molecular details of how flavonoid production in plants is regulated during nodulation have not yet been clarified, but nitrogen availability has been shown to play a role.

## 1. Introduction

Nodulation in legumes evolved as a highly specific interaction between the legumes and gram-negative soil bacteria called rhizobia. The symbiosis is initiated with a chemical signal exchange between host and symbiont. In low nitrogen conditions specific flavonoids are secreted by the host roots, which activate the production of specific lipo-chitooligosaccharide signaling compounds, called Nod factors, by homologous (compatible) rhizobia. Flavonoid perception in the rhizobia is mediated by NodD, a protein that promotes transcription of bacterial *nod* genes involved in synthesis and secretion of Nod factors [1,2]. The perception of specific Nod factors triggers a signaling cascade in the host that leads, in most legumes, to the formation of the specialized intracellular structures called infection threads. The infection thread acts as a conduit to provide access for the rhizobia to the inner root tissues where they are endocytosed into nodule cells and begin to fix nitrogen [3]. While Nod factor recognition is a key determinant of host range specificity [4,5], differences in flavonoid (NodD-mediated) induction of *nod* genes plays an equally important role [6]. Loss of the ability to produce or perceive either Nod factors or flavonoids prevents nodulation [7,8,9,10]. Notably, flavonoids also appear to play a central role in the actinorhizal symbiosis: expression of flavonoid biosynthetic genes is increased in the interaction [11], flavonoids can enhance nodulation [12,13], and the repression of flavonoid production reduces nodulation [14]. This points to a universal role for these compounds in nodulation. As actinorhizal nodulation predates the appearance of legumes this suggests either ancient origins for, or convergent evolution of, the role of flavonoids in nodulation. While it is clear the main role for flavonoids in legume nodulation is to induce Nod factor production by rhizobia, they also regulate other rhizobial responses that are important for symbiosis (reviewed in [15,16] including alterations in growth and motility [17,18]. In the broad host rhizobia NGR234 flavonoids can also induce IAA biosynthesis [19]. While these effects may not be strictly required for the symbiosis in artificial lab conditions their contribution in natural environments should not be underestimated as they may impact on competiveness in the field [20]. In addition to their role in nodulation, secreted flavonoids have other roles in the rhizosphere, particularly in P and Fe acquisition [21,22].

Extensive knowledge of the rhizobial genes responsible for variation in Nod factors has been acquired, for instance galegoid legumes recognize Nod factors that feature alpha-beta-unsaturated fatty acids, while within that group *Medicago* spp. further require that Nod factors be sulphated [23,24,25]. However, even though flavonoids have been studied extensively in legumes, relatively little information is available on which flavonoids play a role in determination of host range. Early work in this area focused on the identification of the key flavonoids being produced and their effects on the rhizobia. The arrival of legume model systems along with new molecular tools offers an opportunity to dissect which flavonoids matter the most in a given interaction and to study when and where they are produced. Research in soybean and *M. truncatula* have highlighted key flavonoids required for the initiation and progression of infection, referred to herein as *infection flavonoids*, as well as a potential role for flavonoids as phytoalexins acting to reinforce specificity in nodulation. In contrast, relatively little progress has been made on the regulation of the production of flavonoids during nodulation. A relationship between flavonoid production and the carbon-nitrogen status is evident, and this may be reflected in flavonoid production in nodules. 

## 2. Flavonoids as Determinants of Host Range

### 2.1. Flavonoids as Infection Signals

**Figure 1:**
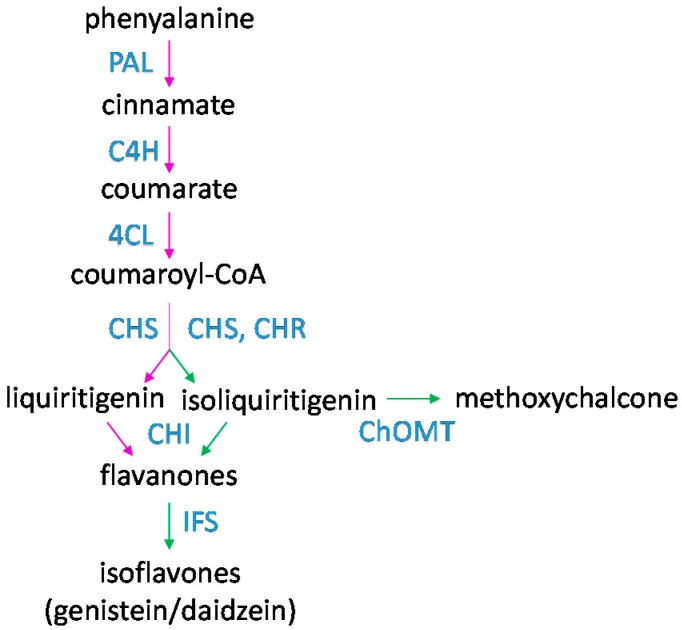
The isoflavonoid biosynthesis pathway. PAL (Phenylalanine ammonia-lyase), C4H (Cinnamate 4-hydroxylase), 4CL (4-coumarate CoA-ligase), CHS (Chalcone synthase), CHR (Chalcone reductase), CHI (Chalcone isomerase), IFS (Isoflavone synthase), ChOMT (Chalcone O-methyltransferase). Legume specific steps are indicated in green.

Flavonoids are low molecular weight secondary metabolites that are produced in plants. They are based upon a fifteen-carbon skeleton consisting of two benzene rings and are biosynthesized by phenylpropanoid pathway. Plants produce a large array of flavonoids. Rosids in particular have undergone a lineage-specific expansion of the *Chalcone synthase* (*CHS*) gene family, which encodes the first committed enzyme of flavonoid biosynthesis, and legumes have had a further expansion of one branch of the *CHS* gene family [26]. Isoflavonoids are a signature characteristic of legumes [27]. The enzymes involved in isoflavonoid synthesis have been identified. Isoliquiritigenin is produced by a legume-specific enzyme, Chalcone reductase (CHR), acting in combination with CHS (reviewed in [28]). The enzyme Chalcone isomerase (CHI) then coverts chalcones to flavanones. Legumes have evolved a novel isoform of CHI that has a preference for isoliquiritigenin as a substrate, in contrast to non-legume CHIs that prefer liquiritigenin. Further action by Isoflavone synthase (IFS) leads to production of isoflavones (a type of isoflavonoids), such as daidzein or genistein (Figure 1). 

The diversity of (iso)flavonoids in legumes appears to be driven in part by the role of these compounds in nodulation. Although legumes produce many flavonoids, only specific subsets have roles in nodulation. To act as nodulation signals flavonoids must be secreted from the roots into the rhizosphere, which includes the root surface and inside infection threads (which are effectively extracellular compartments), where they induce *nod* gene expression [29,30,31,32,33,34,35,36]. The continued induction of the Nod factor biosynthesis operon throughout the infection process is crucial [37,38]. Consequently, the production and release of flavonoids is central to how host-symbiont specificity is achieved. To illustrate this point we’ll consider the flavone luteolin and the chalcone 4, 4′-dihydroxy-2′-methoxychalcone (methoxychalcone) in the *Medicago-Sinorhizobium meliloti* symbiosis. Luteolin is not legume-specific and is found in many plant families [39]. Although it was the first flavonoid identified as a *nod* gene inducer, it can induce *nod* genes across a diverse array of symbionts, including *S. meliloti*, *Rhizobium galegae*, and different subtypes of *R. leguminosarum*, suggesting a lack of specificity [35,40,41]. The non-specific *nod* gene-inducing activity of luteolin is further demonstrated by its ability to activate the NodD of *Mesorhizobium ciceri*, which specifically nodulates chickpea [42]. Furthermore, tests using *M. ciceri* NodD shows it is not activated by alfalfa, pea, and clover root exudates, suggesting that luteolin is not a key *nod* gene-inducer in these species. In fact, although luteolin can induce expression of *S. meliloti* nodulation genes [35] and exogenous application of luteolin can enhance nodulation [43], it has never been detected in *Medicago* root exudates or in nodules [34]. Luteolin is instead secreted in large quantities from germinating seeds, and roles for luteolin as a rhizobial chemoattractant, as well as in biofilm formation and motility, have been proposed [17,44]. 

In contrast with luteolin, methoxychalcone meets most of the criteria for a host infection signal. While many flavonoids are produced in *Medicago* spp., only a few are present in root exudates, and just four are symbiotically induced (Table 1) including methoxychalcone. Methoxychalcone levels are induced by *S. meliloti*, and it is the strongest *nod* gene inducer identified in *Medicago* root exudates having significantly enhanced activity over luteolin [34,45,46]. Methoxychalcone is produced from isoliquiritigenin by the enzyme CHALCONE-O-METHYLTRANSFERASE (ChOMT) and is therefore legume-specific [47,48] (Figure 1). Our recent study has shown that the *M. truncatula* orthologue, *ChOMT1*, and three other close homologues (*ChOMT2*, *ChOMT3*, and *ChOMT4*), were induced in root hairs of rhizobially inoculated plants, and two of these are highly expressed in the infection zone of mature nodules [49,50,51]. Interestingly, although soybean has six *ChOMTs*, none are induced in root hairs during infection by *Bradyrhizobium*, suggesting that production of methoxychalcone is not a general response to rhizobial infection in legumes [52]. Methoxychalcone was also found in *Vicia sativa* root exudates upon rhizobial inoculation and was shown to also have *nod* gene inducing activity with *R. leguminosarum* bv. *viciae*, and *R. leguminosarum* bv. *trifolii*, suggesting that it may have a role in infection in other Trifolieae [53]. Methoxychalcone has also been reported in two other IRLC clade legumes in non-symbiotic contexts [54,55]. Determination of the relative contribution of methoxychalcone to infection and its importance to host range boundaries awaits further studies.

In the soybean-*Bradyrhizobium* symbiosis, genistein and daidzein are proven to be crucial infection signals: they both induce *nod* genes in *B. japonicum* [56,57], they are present in root exudates, and their production is induced by *Bradyrhizobium* and by Nod factors [58]. The most critical evidence is that knockdown of *IFS* greatly reduces the levels of these isoflavonoids and completely blocks nodulation [10]. However, contribution of other related flavonoids cannot be ruled out: genistein is a precursor for prunetin which is symbiotically induced (Table 1; [59]) and is a relatively strong and selective *nod* gene-inducer in *Bradyrhizobium*, activating NodD from *B. japonicum* but not *B. elkanii* [60]. A shared characteristic of these infection flavonoids is that they are symbiotically induced [58]. It is well recognized that rhizobia significantly change the flavonoid profile of their host [61,62,63]), and many of symbiotically up-regulated flavonoids have *nod* gene-inducing activity. Furthermore, these changes in flavonoid composition require that the interaction be compatible (i.e., they are not induced by heterologous rhizobia) and are therefore not part of a general defense response to bacteria, but instead are a hallmark of symbiosis [46,62]. 

Based on this discussion we can define the following key characteristics of infection flavonoids:

- “strong” inducers of *nod* genes in homologous rhizobia
- secreted by roots (i.e., found in root exudate)
- increased biosynthesis in response to rhizobia or Nod factors
- required for rhizobial infection (i.e., genetic evidence)

In other legumes many *nod* gene-inducing flavonoids have been identified (reviewed in [64,65], but only a subset of *nod* gene-inducers are secreted and fewer still are symbiotically enhanced (Table 1). 

The main limitation in identifying infection flavonoids is characterizing their production in the host plants. Genetic evidence implicating specific flavonoids is lacking even in well-established models such as the *Lotus japonicus*-*M. loti* symbiosis. This is partly due to limited knowledge of the flavonoids involved in *nod* gene activation, although some knowledge of exudate components has been obtained from *L. pedunculatus* [69,70]. In other legumes, where this information is available (Table 1), knowledge of the biosynthetic pathways is lacking and genetic resources are limited.

### 2.2. Flavonoid Phytoalexins as Determinants of Host Range

**Figure 2:**
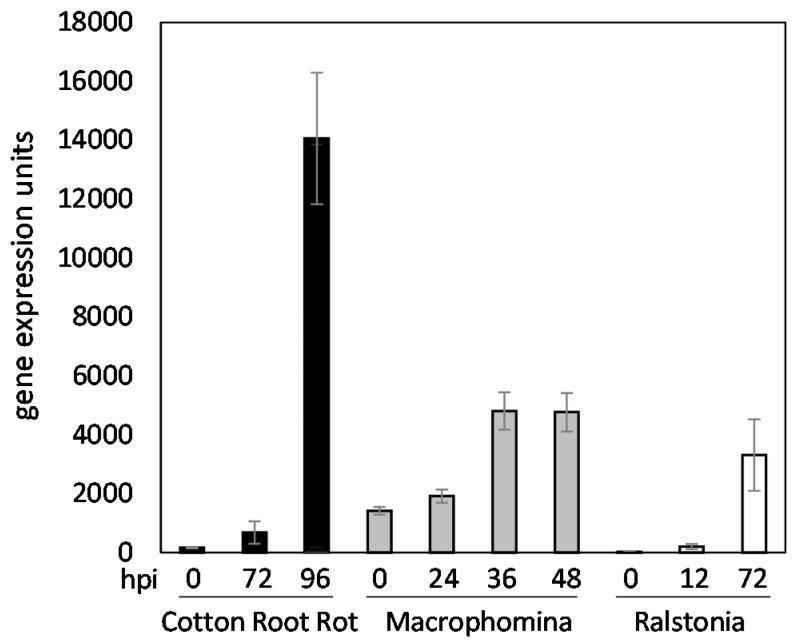
*ChOMT1* expression in pathogen-inoculated roots of *Medicago truncatula*. Data are taken from the Medicago Gene Expression Atlas [79]. Original data for Cotton Root Rot (*Phymatotrichopsis omnivore*) are from Reference [80], and data for *Macrophomina phaseolina* were described by the authors of [81]. Data for *Ralstonia solanacearum* has not been described in a publication. hpi = hours post inoculation. Bars are SD.

Several studies have shown that in addition to *nod* gene-inducing flavonoids the production of phytoalexin flavonoids with anti-bacterial and/or anti-fungal activity is increased during nodulation [63,67,68,71]. The production of phytoalexins during nodulation may at first seem counter-intuitive, but it is clear these phytoalexins are produced during successful interactions and are not part of a generalized defence response to rhizobia. Furthermore many of these have no *nod* gene inducing activity [72], and some, like medicarpin, can antagonize *nod* gene induction [31]. In addition, many *nod* gene-inducing flavonoids are also phytoalexins. For example, methoxychalcone has potent antibacterial activity against gram-positive bacteria [73] and is induced by the elicitor chitosan in pea [74], and genistein has both antifungal and antibacterial activity [75,76]. Furthermore, the *M. truncatula* *ChOMT1* gene is inducible by pathogens, consistent with a role for methoxychalcone as a phytoalexin (Medicago Gene Expression Atlas; Figure 2). The apparently universal role of flavonoids as phytolexins in plants suggests that, along with their role in determining rhizobial host-range, their role in defense was likely a key driver in the expansion and diversification of these compounds in legumes. One phytoalexin, medicarpin, is induced in *S. meliloti-M. truncatula* interactions and by fungal pathogens [67,77,78].

Medicarpin is produced by *Medicago* spp. and other legumes and belongs to a special class of highly diversified isoflavonoid-derived compounds called pterocarpans, including pisatin from pea, and glyceollin from soybean (reviewed in [82,83,84]). Like other isoflavonoids medicarpin is produced through the action of CHR, CHI, and IFS but it additonally requires the action of several other enzymes including VESTITONE REDUCTASE (VR) which catalyses the penultimate step in medicarpin biosynthesis [85,86]. The role of these compounds in the symbiosis has not been clarified, but the finding that the *Medicago* symbiont *S. meliloti*, but not *Bradyrhizobium japonicum* and *M. loti*, is resistant to medicarpin [87], lead to the suggestion of a role for this compound in selection for homologous rhizobia [67]. In support of this idea, recent gene expression studies of *VR* in *M. truncatula* roots revealed increased expression at the sites of rhizobial infection, both in infected root hairs and in the nodule [49,51], suggesting that rhizobia are exposed to medicarpin during infection. Similarly, the soybean symbionts *B. japonicum* and *S. fredii* acquire resistance to glyceollin when exposed to genistein and daidzein [88].

The idea of manipulation of the rhizosphere by the host to favour compatible symbionts has been steadily gaining ground. In *Rhizobium etli*, genes encoding multidrug resistance proteins were identified that conferred resistance to the flavonoids coumarate and naringenin as well as to the pterocarpans phaseollin and phaseollidin; loss of one of these genes led to a 40% reduction of nodulation on *Phaseolus vulgaris* [89]. Similarly, the loss of a multidrug efflux pump component in *B. japonicum* caused a strong decrease in symbiotic nitrogen-fixation activity in soybean, but not in the alternative hosts mung bean and cowpea, suggesting rhizobia have acquired adapatations to specific phytoalexins in host rhizospheres [90]. Other types of compounds will likely play similar roles in rhizobial selection. *Rhizobium* mutants that were susceptible to mimosine, a phytoalexin found in root exudates and nodules of *Mimosa* and *Leucaena* spp., had greatly reduced nodule occupancy on *L. leucocephala* when co-inoculated with the WT strain [91].

### 2.3. Manipulation of Host Range

As discussed above, the two most crucial factors controlling host range are rhizobial Nod factors and the flavonoids that induce their biosynthesis. Knowledge of flavonoid and Nod factor specificities has brought with it the ability to manipulate host range. In soil populations of rhizobia host range barriers can be overcome by lateral transfer of Symbiosis plasmids, in which encode the flavonoid sensor NodD and the Nod factor biosynthesis enzymes for interactions with a specific host [92]. Numerous efforts have shown that transfer of either the *nodD* gene, Nod factor biosynthesis genes or both are sufficent to overcome host-range limits [93], even allowing the pathogen *Agrobacterium tumefaciens* to nodulate some legumes, albeit ineffectively. Perhaps the most impressive effort in this area was by Radutoiu et al. [94], who modified both the symbiont and host to break a host-range barrier. To achieve this, they used *L. japonicus* compatible symbionts carrying a flavonoid-independent NodD activators to nodulate *M. truncatula* roots transgenically expressing the *L. japonicus* Nod factor-receptors. In this case, the flavonoid-independent *M. loti* was able to initiate infection threads and induce underdeveloped nodules on the root, but the infections were mainly arrested in the epidermis, while the flavonoid-independent *R. leguminosarum* strain progressed further into to the nodule and then aborted. It was suggested that the difference in infection progression for the two strains could be due to the relative similarity of the *R. leguminosarum* Nod factor to the *S. meliloti* Nod factor or to differences in surface exopolysaccharides in the strains. Another possibility is that medicarpin, which is known to be toxic to *M. loti* [87] and other phytoalexins such as methoxychalcone, played a role. More studies are needed to better understand the relative contributions of phytoalexins in host range and rhizosphere competition.

## 3. C/N Status May Play a Central Role in the Regulation of Flavonoid Levels in Nodules

**Figure 3:**
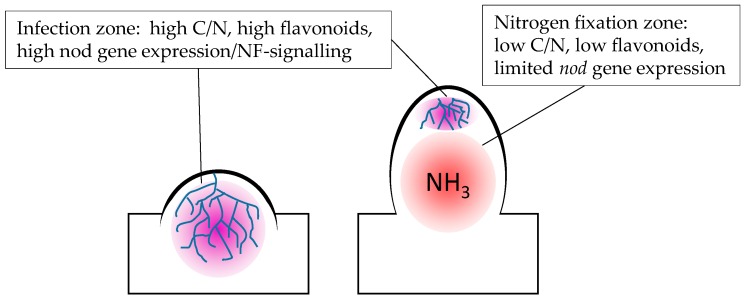
The association between high C/N ratios and flavonoids in nodulation. Areas undergoing infection by rhizobia are dominated by flavonoid-induced Nod factor (NF) signalling and accumulate carbon within amyloplasts. In the bacteroid-containing nitrogen fixation zone, carbon stores have been depleted, flavonoid-related gene expression is low and infection threads are mostly absent.

While much attention in the nodulation field has been focussed on the role of flavonoids, relatively little is known about how their production is regulated. Bhagwat and Thomas (1982) [95] discovered factors in root exudate that promoted nodulation and that could be supressed by the presence of fixed nitrogen. Later, the role of flavonoids in nodulation was revealed and a later study showed that the production of flavonoids is upregulated by low soil nitrogen, which is concordant with the role of flavonoids in nodulation [96]. This relationship between carbon/nitrogen ratios and phenylpropanoid metabolism appears to be a general phenomenon in plants [97,98,99,100,101]. Higher flavonoid levels in the roots, as discussed above, strongly promotes infection through upregulation of *nod* genes and other responses in the rhizobia. Conversely, rhizobial *nod* gene expression is repressed by the presence of ammonium in *S. meliloti* and *B. japonicum* [102,103], reviewed in [104]. These two systems appear, therefore, to act together to regulate infection at different nitrogen availabilities, with the level of available nitrogen controlling plant production of flavonoids but also directly regulating *nod* gene expression in the rhizobia. Fitting with this, the expression of host flavonoid biosynthetic genes and rhizobial *nod* genes is highest in the apex and lowest in the *N*-fixation zone of *M. truncatula* nodules [50]. This increase in flavonoid biosynthesis genes is accompanied by very low nitrate levels in the nodule relative to the root [105], whereas in the nitrogen-fixation zone the expression of flavonoid biosynthetic genes is greatly reduced as is the expression of rhizobial *nod* genes, both potentially a consequence of the ammonia being produced (Figure 3). Indeed, the sensitivity of the *nod* operon to ammonia may explain the near absence of infection threads in the nitrogen fixation zone, while in the infection zone Nod factor signalling induces the production of more flavonoids in a positive feedback loop. The situation in nodule primordia, which is heavily colonized with infection threads but devoid of nitrogen-fixing rhizobia, is similar to that in the nodule apex; in these tissues flavonoid production is high to promote infection (Figure 3; [50]). These observations are circumstantial and require further investigation to determine whether localised nitrogen regulation of host flavonoid synthesis and regulation of *nod* genes by fixed ammonia operate together to define nodule zones. In summary, progress on the regulation of flavonoid production in legumes is limited. In general, the production of flavonoids in plant tissues is stimulated by high C/N ratios, and in legumes low N leads to enhanced secretion of *nod* gene-inducing flavonoids from roots. As flavonoids are critical for rhizobial infection this is likely one of the key mechanisms by which nutrient availability regulates nodulation. This regulation may also be relevant in nodules, where the production of key infection flavonoids appears to be restricted to differentiating tissues and excluded from the nitrogen fixation zone.

## 4. Conclusions and Future Prospects

**Figure 4:**
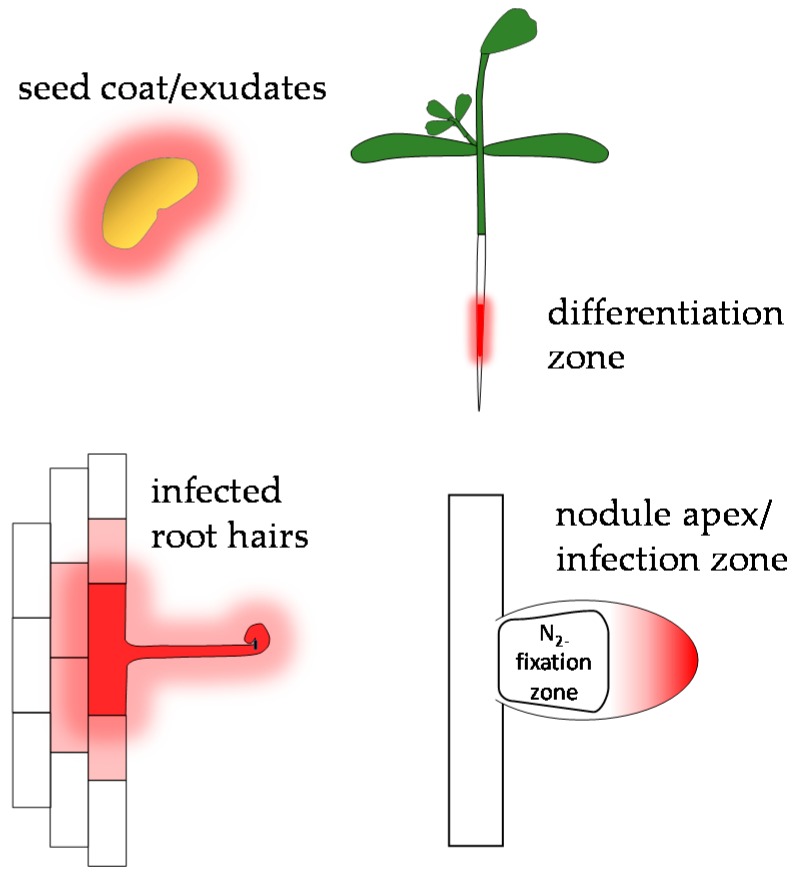
The production and secretion of flavonoids at different stages of growth and development in *Medicago spp.* (**Top left**) Luteolin and other flavonoids are produced in seed coat and are released in the exudate upon imbibition and may play a role in chemoattraction of rhizobia [17,44,45]. (**Top right**) Flavonoids are produced in the root hair elongation zone and some are secreted into the rhizosphere [31,48,106]. (**Bottom left**) *ChOMT* genes are expressed in rhizobially infected root hairs, suggesting that the *nod* gene-inducer methoxychalcone is produced locally [50,51]. (**Bottom right**) *ChOMT* genes are also expressed in the nodule apex/infection zone where infection threads are present, but not in the nitrogen fixation zone [49,50].

Legumes produce a large array of flavonoids in both shoots and roots, and the control of when and where specific flavonoids are secreted is a primary determinant of rhizobial host range, controlling the onset of Nod factor signaling. The requirement for host-range restrictions in the legume-rhizobia symbiosis has given rise to a great diversity of flavonoids and Nod factors of which only a few systems have been studied in detail. Recent genetic studies in model systems indicate that rhizobial infection processes are likely controlled by a limited number of key *nod* gene-inducing flavonoids in each legume. These infection flavonoids are produced locally at infection sites and in nodule primordia and in the infection zone of mature indeterminate nodules, while other flavonoids in seed exudates may play supporting roles (Figure 4). Many of these flavonoids also act as phytoalexins which, along with other symbiosis-induced flavonoids, may have a role in rhizosphere selection of compatible rhizobia and may be important determinants of host range in the field. Since legumes are the third largest plant family, we can predict that the matrix of Nod factor-flavonoid combinations will be immense, providing a rich resource for rhizosphere engineering. However, for this potential to be fully realized more knowledge of specific host determinants is required, particularly the identification of infection flavonoids and the enzymes that produce them, and their corresponding rhizobial NodD proteins.

## Figures and Tables

**Table 1:** Rhizobia and Nod factor-induced flavonoids.

Host Species	(iso)Flavonoids	Tissues	Reference
Soybean	isoliquiritigenin ^1^	root/ root hair	[59]
	liquiritigenin ^2^		
	apigenin		
	prunetin		
	afrormosin		
	amino-flavonoid		
	dihydrokaempferol		
	genkwanin		
	naringenin ^3^		
	biochanin-A ^3^		
	daidzein	root exudates	[58]
	genistein		
	coumestrol		
*Phaseolus vulgaris*	genistein	root exudates	[66]
	daidzein		[63]
	coumestrol		
	isoliquiritigenin		
	naringenin		
	liquiritigenin		
*Medicago sativa*	methoxychalcone	root exudates	[67]
	formononetin ^3^		
	medicarpin ^2^		
*Vicia sativa*	methoxychalcone	root exudates	[46]
	isoliquiritigenin		
	liquiritigenin		
	hesperitin		
	naringenin		
	7,3′-dihydroxy-4′-methoxyflavanone		
	7,4′-dihydroxy-3′-methoxyflavanone		
	5,7,4′-trihydroxy-3′-methoxyflavanone		
*Trifolium subterraneum*	4′,7-dihydroxyflavone	root exudates	[61]
*Pisum sativum*	pisatin	root exudate	[68]

^1^ bold indicates are *nod* gene inducers; ^2^ glycoside also detected ^3^ only glycoside detected.

## References

[1] Spaink H.P., Okker R.J.H., Wijffelman C.A., Tak T., Goosenderoo L., Pees E., Vanbrussel A.A.N., Lugtenberg B.J.J. (1989). Symbiotic properties of rhizobia containing a flavonoid-independent hybrid nodD product. J. Bacteriol..

[2] Mulligan J.T., Long S.R. (1985). Induction of *Rhizobium meliloti*
*nodC* expression by plant exudate requires nodD. Proc. Natl. Acad. Sci. USA.

[3] Oldroyd G.E.D., Murray J.D., Poole P.S., Downie J.A. (2011). The rules of engagement in the legume*-*rhizobial Symbiosis. Annu. Rev. Genet..

[4] Lerouge P., Roche P., Faucher C., Maillet F., Truchet G., Prome J.C., Denarie J. (1990). Symbiotic host-specificity of *Rhizobium-meliloti* is determined by a sulfated and acylated glucosamine oligosaccharide signal. Nature.

[5] Spaink H.P., Sheeley D.M., Vanbrussel A.A.N., Glushka J., York W.S., Tak T., Geiger O., Kennedy E.P., Reinhold V.N., Lugtenberg B.J.J. (1991). A novel highly unsaturated fatty-acid moiety of lipo-oligosaccharide signals determines host specificity of rhizobium. Nature.

[6] Spaink H.P., Wijffelman C.A., Pees E., Okker R.J.H., Lugtenberg B.J.J. (1987). *Rhizobium* nodulation gene *nodD* as a determinant of host specificity. Nature.

[7] Radutoiu S., Madsen L.H., Madsen E.B., Felle H.H., Umehara Y., Gronlund M., Sato S., Nakamura Y., Tabata S., Sandal N. (2003). Plant recognition of symbiotic bacteria requires two LysM receptor-like kinases. Nature.

[8] Madsen E.B., Madsen L.H., Radutoiu S., Olbryt M., Rakwalska M., Szczyglowski K., Sato S., Kaneko T., Tabata S., Sandal N. (2003). A receptor kinase gene of the LysM type is involved in legume perception of rhizobial signals. Nature.

[9] Wasson A.P., Pellerone F.I., Mathesius U. (2006). Silencing the flavonoid pathway in Medicago truncatula inhibits root nodule formation and prevents auxin transport regulation by rhizobia. Plant Cell.

[10] Subramanian S., Stacey G., Yu O. (2006). Endogenous isoflavones are essential for the establishment of symbiosis between soybean and *Bradyrhizobium japonicum*. Plant J..

[11] Auguy F., Abdel-Lateif K., Doumas P., Badin P., Guerin V., Bogusz D., Hocher V. (2011). Activation of the isoflavonoid pathway in actinorhizal symbioses. Funct. Plant Biol..

[12] Popovici J., Comte G., Bagnarol E., Alloisio N., Fournier P., Bellvert F., Bertrand C., Fernandez M.P. (2010). Differential effects of rare specific flavonoids on compatible and incompatible strains in the *Myrica gale*-Frankia actinorhizal symbiosis. Appl. Environ. Microbiol..

[13] Popovici J., Walker V., Bertrand C., Bellvert F., Fernandez M.P., Comte G. (2011). Strain specificity in the *Myricaceae-Frankia* symbiosis is correlated to plant root phenolics. Funct. Plant Biol..

[14] Abdel-Lateif K., Vaissayre V., Gherbi H., Verries C., Meudec E., Perrine-Walker F., Cheynier V., Svistoonoff S., Franche C., Bogusz D. (2013). Silencing of the chalcone synthase gene in *Casuarina glauca* highlights the important role of flavonoids during nodulation. New Phytol..

[15] Hassan S., Mathesius U. (2012). The role of flavonoids in root-rhizosphere signalling: Opportunities and challenges for improving plant-microbe interactions. J. Exp. Bot..

[16] Weston L.A., Mathesius U. (2013). Flavonoids: Their structure, biosynthesis and role in the rhizosphere, including allelopathy. J. Chem. Ecol..

[17] Caetanoanolles G., Cristestes D.K., Bauer W.D. (1988). Chemotaxis of *Rhizobium-meliloti* to the plant flavone luteolin requires functional nodulation genes. J. Bacteriol..

[18] Aguilar J.M.M., Ashby A.M., Richards A.J.M., Loake G.J., Watson M.D., Shaw C.H. (1988). Chemotaxis of *Rhizobium-leguminosarum* biovar phaseoli towards flavonoid inducers of the symbiotic nodulation genes. J. Gen. Microbiol..

[19] Theunis M., Kobayashi H., Broughton W.J., Prinsen E. (2004). Flavonoids, nodD1, nodD2, and nod-box NB15 modulate expression of the y4wEFG locus that is required for indole-3-acetic acid synthesis in *Rhizobium* sp. strain NGR234. Mol. Plant-Microbe Interact..

[20] Maj D., Wielbo J., Marek-Kozaczuk M., Skorupska A. (2010). Response to flavonoids as a factor influencing competitiveness and symbiotic activity of *Rhizobium leguminosarum*. Microbiol. Res..

[21] Cesco S., Mimmo T., Tonon G., Tomasi N., Pinton R., Terzano R., Neumann G., Weisskopf L., Renella G., Landi L. (2012). Plant-borne flavonoids released into the rhizosphere: Impact on soil bio-activities related to plant nutrition. A review. Biol. Fertil. Soils.

[22] Tomasi N., Weisskopf L., Renella G., Landi L., Pinton R., Varanini Z., Nannipieri P., Torrent J., Martinoia E., Cesco S. (2008). Flavonoids of white lupin roots participate in phosphorus mobilization from soil. Soil Biol. Biochem..

[23] Faucher C., Maillet F., Vasse J., Rosenberg C., Vanbrussel A.A.N., Truchet G., Denarie J. (1988). *Rhizobium-meliloti* host range nodh-gene determines production of an alfalfa-specific extracellular signal. J. Bacteriol..

[24] Debelle F., Moulin L., Mangin B., Denarie J., Boivin C. (2001). NoD genes and nod signals and the evolution of the *Rhizobium* legume symbiosis. Acta Biochim. Pol..

[25] Wais R.J., Keating D.H., Long S.R. (2002). Structure-function analysis of nod factor-induced root hair calcium spiking in *Rhizobium-Legume Symbiosis*. Plant Physiol..

[26] Zavala K., Opazo J.C. (2015). Lineage-specific expansion of the *chalcone synthase* gene family in rosids. PLoS ONE.

[27] Dewick P.M., Harborne J.B. (1988). Isoflavonoids. The Flavonoids: Advances in Research Since 1980.

[28] Wang X. (2011). Structure, function, and engineering of enzymes in isoflavonoid biosynthesis. Funct. Integr. Genom..

[29] Subramanian S., Stacey G., Yu O. (2007). Distinct, crucial roles of flavonoids during legume nodulation. Trends Plant Sci..

[30] Chovanec P., Novak K. (2005). Visualization of nodulation gene activity on the early stages of *Rhizobium leguminosarum* bv. *Viciae symbiosis*. Folia Microbiol..

[31] Zuanazzi J.A.S., Clergeot P.H., Quirion J.C., Husson H.P., Kondorosi A., Ratet P. (1998). Production of *Sinorhizobium meliloti*
*nod* gene activator and repressor flavonoids from *Medicago sativa* roots. Mol. Plant-Microbe Interact..

[32] Phillips D.A., Dakora F.D., Sande E., Joseph C.M., Zon J. (1994). Synthesis, release, and transmission of alfalfa signals to rhizobial symbionts. Plant Soil.

[33] Kape R., Parniske M., Brandt S., Werner D. (1992). Isoliquiritigenin, a strong *nod* gene-inducing and glyceollin resistance-inducing flavonoid from soybean root exudate. Appl. Environ. Microbiol..

[34] Maxwell C.A., Hartwig U.A., Joseph C.M., Phillips D.A. (1989). A chalcone and two related flavonoids released from alfalfa roots induce *nod* genes of *Rhizobium-meliloti*. Plant Physiol..

[35] Peters N.K., Frost J.W., Long S.R. (1986). A plant flavone, luteolin, induces expression of *Rhizobium-meliloti* nodulation genes. Science.

[36] Redmond J.W., Batley M., Djordjevic M.A., Innes R.W., Kuempel P.L., Rolfe B.G. (1986). Flavones induce expression of nodulation genes in *Rhizobium*. Nature.

[37] Marie C., Barny M.A., Downie J.A. (1992). *Rhizobium leguminosarum* has two glucosamine synthases, glms and nodm, required for nodulation and development of nitrogen-fixing nodules. Mol. Microbiol..

[38] Den Herder J., Vanhee C., De Rycke R., Corich V., Holsters M., Goormachtig S. (2007). Nod factor perception during infection thread growth fine-tunes nodulation. Mol. Plant-Microbe Interact..

[39] Bisby F. (1994). Phytochemical Dictionary of the Leguminosae.

[40] Zaat S.A.J., Wijffelman C.A., Spaink H.P., Vanbrussel A.A.N., Okker R.J.H., Lugtenberg B.J.J. (1987). Induction of the *nodA* promoter of *Rhizobium leguminosarum* Sym plasmid pRL1JI by plant flavanones and flavones. J. Bacteriol..

[41] Suominen L., Luukkainen R., Roos C., Lindstrom K. (2003). Activation of the *nodA* promoter by the *nodD* genes of *Rhizobium galegae* induced by synthetic flavonoids or *Galega orientalis* root exudate. FEMS Microbiol. Lett..

[42] Kamboj D.V., Bhatia R., Pathak D.V., Sharma P.K. (2010). Role of nodD gene product and flavonoid interactions in induction of nodulation genes in *Mesorhizobium ciceri*. Physiol. Mol. Biol. Plants.

[43] Kapulnik Y., Joseph C.M., Phillips D.A. (1987). Flavone limitations to root nodulation and symbiotic *Nitrogen-fixation* in alfalfa. Plant Physiol..

[44] Spini G., Decorosi F., Cerboneschi M., Tegli S., Mengoni A., Viti C., Giovannetti L. (2016). Effect of the plant flavonoid luteolin on *Ensifer meliloti* 3001 phenotypic responses. Plant Soil.

[45] Hartwig U.A., Maxwell C.A., Joseph C.M., Phillips D.A. (1990). Chrysoeriol and luteolin released from alfalfa seeds induce *nod* genes in *Rhizobium meliloti*. Plant Physiol..

[46] Recourt K., Schripsema J., Kijne J.W., Vanbrussel A.A.N., Lugtenberg B.J.J. (1991). Inoculation of *Vicia-sativa* subsp nigra roots with *Rhizobium-leguminosarum* biovar viciae results in release of *nod* gene activating flavanones and chalcones. Plant Mol. Biol..

[47] Maxwell C.A., Edwards R., Dixon R.A. (1992). Identification, purification, and characterization of s-adenosyl-l-methionine-isoliquiritigenin 2′-O-methyltransferase from alfalfa (*Medicago sativa* L.). Arch. Biochem. Biophys..

[48] Maxwell C.A., Harrison M.J., Dixon R.A. (1993). Molecular characterization and expression of alfalfa isoliquiritigenin 2′-O-methyltransferase, an enzyme specifically involved in the biosynthesis of an inducer of *Rhizobium meliloti* nodulation genes. Plant J..

[49] Roux B., Rodde N., Jardinaud M.-F., Timmers T., Sauviac L., Cottret L., Carrere S., Sallet E., Courcelle E., Moreau S. (2014). An integrated analysis of plant and bacterial gene expression in symbiotic root nodules using laser-capture microdissection coupled to rna sequencing. Plant J..

[50] Chen D.-S., Liu C.-W., Roy S., Cousins D., Stacey N., Murray J.D. (2015). Identification of a core set of rhizobial infection genes using data from single cell-types. Front. Plant Sci..

[51] Breakspear A., Liu C., Roy S., Stacey N., Rogers C., Trick M., Morieri G., Mysore K.S., Wen J., Oldroyd G.E.D. (2014). The root hair “infectome” of *Medicago truncatula* uncovers changes in cell cycle genes and reveals a requirement for auxin signaling in rhizobial infection. Plant Cell.

[52] Libault M., Farmer A., Brechenmacher L., Drnevich J., Langley R.J., Bilgin D.D., Radwan O., Neece D.J., Clough S.J., May G.D. (2010). Complete transcriptome of the soybean root hair cell, a single-cell model, and its alteration in response to *Bradyrhizobium japonicum* infection. Plant Physiol..

[53] Recourt K., van Tunen A.J., Mur L.A., van Brussel A.A., Lugtenberg B.J., Kijne J.W. (1992). Activation of flavonoid biosynthesis in roots of *Vicia sativa* subsp. *Nigra* plants by inoculation with *Rhizobium leguminosarum* biovar viciae. Plant Mol. Biol..

[54] Ayabe S.I., Kobayashi M., Hikichi M., Matsumoto K., Furuya T. (1980). Flavonoids from the cultured-cells of *Glycyrrhiza echinata*. Phytochemistry.

[55] Carlson R.E., Dolphin D.H. (1982). *Pisum-sativum* stress metabolites-2 cinnamylphenols and a 2′-methoxychalcone. Phytochemistry.

[56] Kosslak R.M., Bookland R., Barkei J., Paaren H.E., Appelbaum E.R. (1987). Induction of *Bradyrhizobium japonicum* common *nod* genes by isoflavones isolated from *Glycine max*. Proc. Natl. Acad. Sci. USA.

[57] Pueppke S.G., Bolanos-Vasquez M.C., Werner D., Bec-Ferte M.P., Prome J.C., Krishnan H.B. (1998). Release of flavonoids by the soybean cultivars mccall and peking and their perception as signals by the nitrogen-fixing symbiont *Sinorhizobium fredii*. Plant Physiol..

[58] Schmidt P.E., Broughton W.J., Werner D. (1994). Nod factors of *Bradyrhizobium-japonicum* and *Rhizobium* sp. NGR234 induce flavonoid accumulation in soybean root exudate. Mol. Plant-Microbe Interact..

[59] Brechenmacher L., Lei Z., Libault M., Findley S., Sugawara M., Sadowsky M.J., Sumner L.W., Stacey G. (2010). Soybean metabolites regulated in root hairs in response to the symbiotic bacterium *Bradyrhizobium*
*japonicum*. Plant Physiol..

[60] Yokoyama T. (2008). Flavonoid-responsive NodY-lacZ expression in three phylogenetically different *Bradyrhizobium* groups. Can. J. Microbiol..

[61] Lawson C.G.R., Rolfe B.G., Djordjevic M.A. (1996). *Rhizobium* inoculation induces condition-dependent changes in the flavonoid composition of root exudates from *Trifolium subterraneum*. Aust. J. Plant Physiol..

[62] Vanbrussel A.A.N., Recourt K., Pees E., Spaink H.P., Tak T., Wijffelman C.A., Kijne J.W., Lugtenberg B.J.J. (1990). A biovar-specific signal of *Rhizobium-leguminosarum* bv viciae induces increased nodulation gene-inducing activity in root exudate of *Vicia sativa* subsp *nigra*. J. Bacteriol..

[63] Dakora F.D., Joseph C.M., Phillips D.A. (1993). Common bean root exudates contain elevated levels of daidzein and coumestrol in response to *Rhizobium* inoculation. Mol. Plant-Microbe Interact..

[64] Janczarek M., Rachwal K., Marzec A., Grzadziel J., Palusinska-Szysz M. (2015). Signal molecules and cell-surface components involved in early stages of the legume*-Rhizobium* interactions. Appl. Soil Ecol..

[65] Cooper J.E. (2007). Early interactions between legumes and rhizobia: Disclosing complexity in a molecular dialogue. J. Appl. Microbiol..

[66] BolanosVasquez M.C., Warner D. (1997). Effects of *Rhizobium tropici*, *R. etli*, and *R. leguminosarum* bv *phaseoli* on *nod* gene-inducing flavonoids in root exudates of *Phaseolus vulgaris*. Mol. Plant-Microbe Interact..

[67] Dakora F.D., Joseph C.M., Phillips D.A. (1993). Alfalfa (*Medicago sativa* l) root exudates contain isoflavonoids in the presence of *Rhizobium meliloti*. Plant Physiol..

[68] Novak K., Lisa L., Skrdleta V. (2004). Rhizobial *nod* gene-inducing activity in pea nodulation mutants: Dissociation of nodulation and flavonoid response. Physiol. Plant.

[69] Cooper J.E., Rao J.R. (1992). Localized changes in flavonoid biosynthesis in roots of *Lotus pedunculatus* after infection by *Rhizobium loti*. Plant Physiol..

[70] Steele H.L., Werner D., Cooper J.E. (1999). Flavonoids in seed and root exudates of *Lotus pedunculatus* and their biotransformation by *Mesorhizobium loti*. Physiol. Plant.

[71] Parniske M., Zimmermann C., Cregan P.B., Werner D. (1990). Hypersensitive reaction of nodule cells in the *Glycine* sp./*Bradyrhizobium japonicum* symbiosis occurs at the genotype-specific level. Bot. Acta.

[72] Novak K., Kropacova M., Havlicek V., Skrdleta V. (1995). Isoflavonoid phytoalexin pisatin is not recognized by the flavonoid receptor nodD of *Rhizobium leguminosarum* bv viciae. Folia Microbiol..

[73] Haraguchi H., Tanimoto K., Tamura Y., Mizutani K., Kinoshita T. (1998). Mode of antibacterial action of retrochalcones from *Glycyrrhiza inflata*. Phytochemistry.

[74] Akiyama K., Kawazu K., Kobayashi A. (1994). Partially n-deacetylated chitin elicitor induces antimicrobial flavonoids in pea epicotyls. Z. Fur Naturforschung C.

[75] Ulanowska K., Tkaczyk A., Konopa G., Wegrzyn G. (2006). Differential antibacterial activity of genistein arising from global inhibition of DNA, rna and protein synthesis in some bacterial strains. Arch. Microbiol..

[76] Weidenborner M., Hindorf H., Jha H.C., Tsotsonos P., Egge H. (1990). Antifungal activity of isoflavonoids in different reduced stages on *Rhizoctonia-solani* and *Sclerotium-rolfsii*. Phytochemistry.

[77] Paiva N.L., Oommen A., Harrison M.J., Dixon R.A. (1994). Regulation of isoflavonoid metabolism in alfalfa. Plant Cell Tissue Organ Cult..

[78] Guenoune D., Galili S., Phillips D.A., Volpin H., Chet I., Okon Y., Kapulnik Y. (2001). The defense response elicited by the pathogen *Rhizoctonia solani* is suppressed by colonization of the am-fungus *Glomus intraradices*. Plant Sci..

[79] Benedito V.A., Torres-Jerez I., Murray J.D., Andriankaja A., Allen S., Kakar K., Wandrey M., Verdier J., Zuber H., Ott T. (2008). A gene expression atlas of the model legume *Medicago truncatula*. Plant J..

[80] Uppalapati S.R., Marek S.M., Lee H.K., Nakashima J., Tang Y., Sledge M.K., Dixon R.A., Mysore K.S. (2009). Global gene expression profiling during *Medicago truncatula*-*Phymatotrichopsis*
*omnivora* interaction reveals a role for jasmonic acid, ethylene, and the flavonoid pathway in disease development. Mol. Plant Microbe Interact..

[81] Mah K.M., Uppalapati S.R., Tang Y.H., Allen S., Shuai B. (2012). Gene expression profiling of *Macrophomina phaseolina* infected *Medicago truncatula* roots reveals a role for auxin in plant tolerance against the charcoal rot pathogen. Physiol. Mol. Plant Pathol..

[82] Dakora F.D., Phillips D.A. (1996). Diverse functions of isoflavonoids in legumes transcend anti-microbial definitions of phytoalexins. Physiol. Mol. Plant Pathol..

[83] Farag M.A., Huhman D.V., Lei Z., Sumner L.W. (2007). Metabolic profiling and systematic identification of flavonoids and isoflavonoids in roots and cell suspension cultures of *Medicago truncatula* using HPLC-UV-ESI-MS and GC-MS. Phytochemistry.

[84] Hargreaves J.A., Mansfield J.W., Coxon D.T. (1976). Identification of medicarpin as a phytoalexin in broad bean plant (*Vicia-faba-L*). Nature.

[85] Guo L., Dixon R.A., Paiva N.L. (1994). Conversion of vestitone to medicarpin in alfalfa (*Medicago sativa* L.) is catalyzed by two independent enzymes. Identification, purification, and characterization of vestitone reductase and 7,2′-dihydroxy-4′-methoxyisoflavanol dehydratase. J. Biol. Chem..

[86] Guo L., Paiva N.L. (1995). Molecular cloning and expression of alfalfa (*Medicago sativa* L.) vestitone reductase, the penultimate enzyme in medicarpin biosynthesis. Arch. Biochem. Biophys..

[87] Pankhurst C.E., Biggs D.R. (1980). Sensitivity of *Rhizobium* to selected isoflavonoids. Can. J. Microbiol..

[88] Parniske M., Ahlborn B., Werner D. (1991). Isoflavonoid-inducible resistance to the phytoalexin glyceollin in soybean rhizobia. J. Bacteriol..

[89] Gonzalez-Pasayo R., Martinez-Romero E. (2000). Multiresistance genes of *Rhizobium etli* CFN42. Mol. Plant-Microbe Interact..

[90] Lindemann A., Koch M., Pessi G., Muller A.J., Balsiger S., Hennecke H., Fischer H.M. (2010). Host-specific symbiotic requirement of BdeAB, a RegR-controlled RND-type efflux system in *Bradyrhizobium japonicum*. FEMS Microbiol. Lett..

[91] Soedarjo M., Borthakur D. (1998). Mimosine, a toxin produced by the tree-legume leucaena provides a nodulation competition advantage to mimosine-degrading rhizobium strains. Soil Biol. Biochem..

[92] Broughton W.J., Samrey U., Stanley J. (1987). Ecological genetics of *Rhizobium meliloti*-*Symbiotic* plasmid transfer in the *Medicago sativa* rhizosphere. FEMS Microbiol. Lett..

[93] Espuny M.R., Ollero F.J., Bellogin R.A., Ruizsainz J.E., Perezsilva J. (1987). Transfer of the *Rhizobium-leguminosarum* biovar *trifolii* symbiotic plasmid prtr5a to a strain of rhizobium sp. that nodulates on hedysarum-coronarium. J. Appl. Bacteriol..

[94] Radutoiu S., Madsen L.H., Madsen E.B., Jurkiewicz A., Fukai E., Quistgaard E.M., Albrektsen A.S., James E.K., Thirup S., Stougaard J. (2007). LysM domains mediate *Lipochitin-oligosaccharide* recognition and nfr genes extend the symbiotic host range. EMBO J..

[95] Bhagwat A.A., Thomas J. (1982). *Legume-rhizobium* interactions-cowpea root exudate elicits faster nodulation response by rhizobium species. Appl. Environ. Microbiol..

[96] Coronado C., Zuanazzi J.A.S., Sallaud C., Quirion J.C., Esnault R., Husson H.P., Kondorosi A., Ratet P. (1995). Alfalfa root flavonoid production is nitrogen regulated. Plant Physiol..

[97] Wan H., Zhang J., Song T., Tian J., Yao Y. (2015). Promotion of flavonoid biosynthesis in leaves and calli of ornamental crabapple (*Malus* sp.) by high carbon to nitrogen ratios. Front. Plant Sci..

[98] Fritz C., Palacios-Rojas N., Feil R., Stitt M. (2006). Regulation of secondary metabolism by the carbon-nitrogen status in tobacco: Nitrate inhibits large sectors of phenylpropanoid metabolism. Plant J..

[99] Solfanelli C., Poggi A., Loreti E., Alpi A., Perata P. (2006). Sucrose-specific induction of the anthocyanin biosynthetic pathway in arabidopsis. Plant Physiol..

[100] Lea U.S., Slimestad R., Smedvig P., Lillo C. (2007). Nitrogen deficiency enhances expression of specific myb and bhlh transcription factors and accumulation of end products in the flavonoid pathway. Planta.

[101] Bonguebartelsman M., Phillips D.A. (1995). Nitrogen stress regulates gene-expression of enzymes in the flavonoid biosynthetic-pathway of tomato. Plant Physiol. Biochem..

[102] Dusha I., Bakos A., Kondorosi A., Debruijn F.J., Schell J. (1989). The *Rhizobium-meliloti* early nodulation genes (nodabc) are nitrogen-regulated—Isolation of a mutant strain with efficient nodulation capacity on alfalfa in the presence of ammonium. Mol. Gen. Genet..

[103] Wang S.P., Stacey G. (1990). Ammonia regulation of *nod* genes in *Bradyrhizobium-japonicum*. Mol. Gen. Genet..

[104] Dusha I. (2002). Nitrogen control of bacterial signal production in *Rhizobium meliloti*—Alfalfa symbiosis. Indian J. Exp. Biol..

[105] Hunter W.J. (1983). Soybean root and nodule nitrate reductase. Physiol. Plant..

[106] Peters N.K., Long S.R. (1988). Alfalfa root exudates and compounds which promote or inhibit induction of *Rhizobium-meliloti* nodulation genes. Plant Physiol..

